# Antioxidant Properties and the Formation of Iron Coordination Complexes of 8-Hydroxyquinoline

**DOI:** 10.3390/ijms19123917

**Published:** 2018-12-07

**Authors:** Vladimir Chobot, Franz Hadacek, Gert Bachmann, Wolfram Weckwerth, Lenka Kubicova

**Affiliations:** 1 Division of Molecular Systems Biology, Department of Ecogenomics and Systems Biology, Faculty of Life Sciences, University of Vienna, Althanstrasse 14, A-1090 Vienna, Austria; gert.bachmann@univie.ac.at (G.B.); wolfram.weckwerth@univie.ac.at (W.W.); lenka.kubicova@univie.ac.at (L.K.); 2 Department of Plant Biochemistry, Albrecht-von-Haller Institut, Georg-August-Universität Göttingen, Justus-von-Liebig-Weg 11, D-37077 Göttingen, Germany; franz.hadacek@biologie.uni-goettingen.de

**Keywords:** Alzheimer’s, Fenton reaction, hydroxyl radical, iron chelates, reactive oxygen species

## Abstract

Background: The alkaloid 8-hydroxyquinoline (8HQ) is well-known for various biological activities, including antioxidant effects and especially for the formation of coordination complexes with various transition metals, such as iron, amongst others. Therefore, 8HQ was extensively explored as a promising antineurodegenerative agent. However, other authors noted pro-oxidant effects of 8HQ. Here, we explore the pro- and antioxidant properties of 8HQ, especially in context of coordination complexes with iron (II) and iron (III). Methods: Nano-electrospray−mass spectrometry, differential pulse voltammetry, deoxyribose degradation, iron (II) autoxidation, and brine shrimp mortality assays were used. Results: 8HQ formed a complex mixture of coordination complexes with iron (II) and iron (III). Furthermore, 8HQ showed antioxidant effects but no pro-oxidant ones. In the brine shrimp mortality assay, 8HQ demonstrated toxicity that decreased in the presence of iron (III). Conclusions: 8HQ is a potent antioxidant whose effects depend not only on the formation of the coordination complexes with iron ions, but surely on the scavenging activities due to the redox properties of the 8-hydroxyl group. No pro-oxidant effects were observed in the set of the used assays.

## 1. Introduction

8-Hydroxyquinoline (oxine, 8-quinolinol, 8HQ) is an alkaloid with a rather simple chemical structure and occurs in plants of families *Asteraceae* [1] and *Euphorbiaceae* [2]. Previous studies revealed a very large spectrum of various biological activities, such as phytotoxic [1,3], antibacterial [4,5], antifungal [6], and insecticidal [2] activities for 8HQ and structurally related compounds. In recent decades, however, its potential as a cure for some neurodegenerative diseases such as Alzheimer’s and Parkinson’s diseases raised attention [7,8]. Beneficial effects in this context have been attributed to the antioxidant properties of 8HQ [9].

The curative effects of 8HQ depend primarily on direct scavenging and also on its ability to form coordination complexes with transition metals such as iron [10]. These metals can accumulate in the brain tissue under pathological conditions, and can accelerate tissue destruction by catalyzing the formation of cytotoxic reactive oxygen species (ROS), especially hydroxyl radicals [11].

The antioxidant activities of 8HQ have been extensively investigated for a long time. However, the different explorations procured contradictory results in terms of the pro- and antioxidant properties of 8HQ [8,10,12]. Several authors reported ROS generation and cytotoxicity as a consequence of pro-oxidant effects of Fe−8HQ coordination complexes in vitro [12,13] as well as in cell culture [14] experiments. In contrast, other authors suggested that the antioxidant and beneficial effects of 8HQ and its derivatives in neurodegenerative or other diseases associated with accumulation of “poorly liganded” iron are connected primarily with coordination complex formation [9,11,15]. Especially, Kayyali et al. characterized 8HQ as a potent iron (III) complexation agent but poor direct ROS scavenger. However, in other experimental arrangements, 8HQ was proved as an effective direct ROS scavenger [10].

Furthermore, although 8HQ is used in analytical chemistry for its capability of forming coordination complexes with transition metals [16], the composition of Fe−8HQ coordination complexes in situ was not fully described. Therefore, we explored the coordination complex formation ability of 8-hydroxyquinoline with iron, their pro- and antioxidant properties, and their toxic effects.

For this purpose, we used nano-ESI−MS and a set of well-established assays: deoxyribose degradation [17], metal autoxidation [18], and brine shrimp (*Artemia salina* L.) mortality [19,20]. The deoxyribose degradation assays explore the ability of the tested substances to affect hydroxyl radical production by the transition metal-catalyzed Fenton reaction. The metal autoxidation assay assesses the efficiency of the tested compound to influence the metal redox properties in terms of molecular oxygen reduction to ROS. The toxicity of 8HQ and its coordination complexes with iron was studied by examining its effects on brine shrimp mortality—an assay that is commonly used for screening biological activities [20].

## 2. Results

### 2.1. Mass Spectrometry

**Figure 1:**
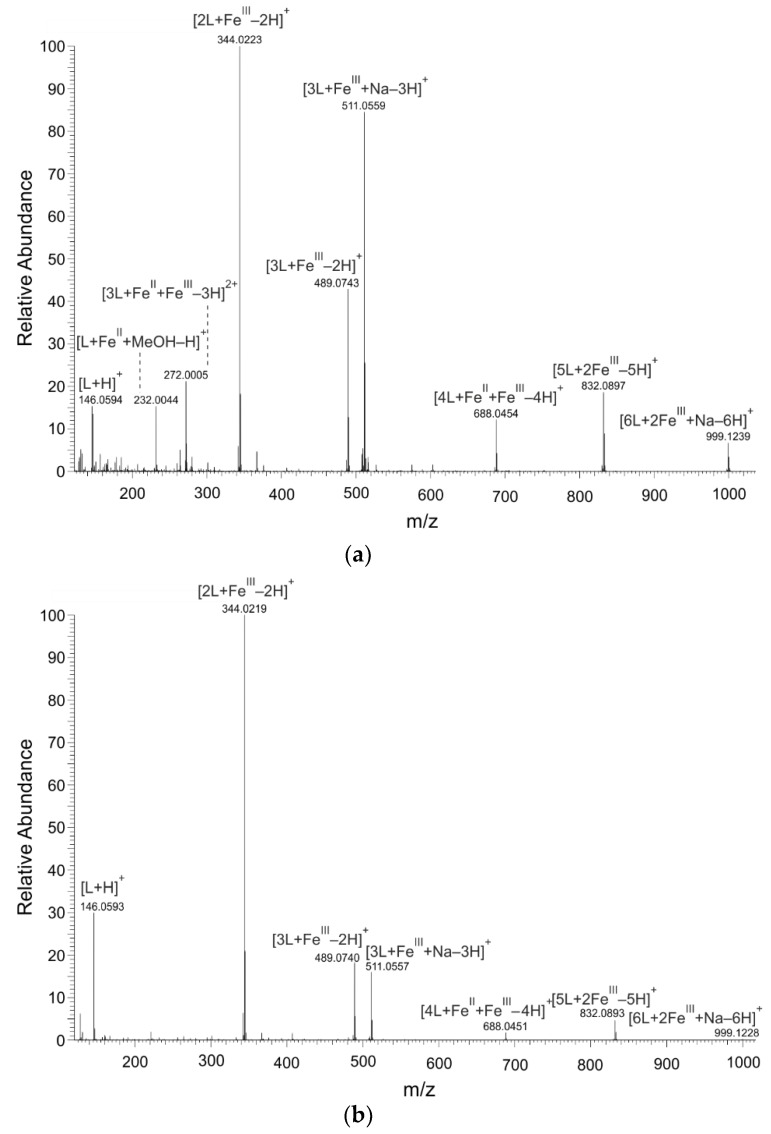
Mass spectra of Fe−8HQ (L) coordination complexes detected in solutions by nano-ESI−MS (nano-electrospray−mass spectrometry); positive ionization mode. The solutions were prepared by mixing of 8-hydroxyquinoline (8HQ) solution with (**a**) Fe^II^ or (**b**) Fe^III^ solutions in molar ratio of metal to ligand of 1:2.

The MS spectra proved that 8HQ formed coordination complexes with both valence forms of iron ions: iron (II) and iron (III) (Figure 1). The isotopic pattern of detected complexes corresponded to the characteristic isotopic pattern of iron (^54^Fe 5.8%, ^56^Fe 91.7%, ^57^Fe 2.2%, and ^58^Fe 0.3%). Figure 1 and Table 1 present the assumed compositions and *m*/*z* values of prominent coordination complexes of the ^56^Fe isotope. The figure and the table point out results of the analyzed solutions in which the molar ratio of metal to ligand was adjusted to 1:2. In solutions with other metal-to-ligand ratios, only the intensity but not the *m*/*z* values significantly differed.

### 2.2. Differential Pulse Voltammetry

**Figure 2:**
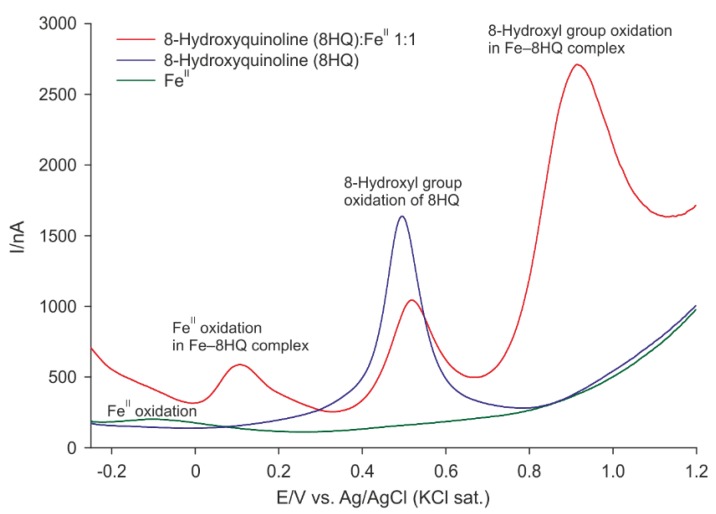
Differential pulse voltammograms of a 1:1 Fe^II^−8HQ mixture, Fe^II^, and 8HQ solutions.

The differential pulse voltammogram of 8HQ showed one prominent peak at 482 mV (Figure 2). The shape of this peak indicated a complicated electrochemical oxidation process of the 8-hydroxyl group that is probably followed by further chemical reactions as proposed by Stevic et al. [21]. After the addition of iron (II) solution, the current of this peak decreased (Figure 2). Furthermore, its potential shifted to the anodic direction (518 mV). Moreover, two other peaks at 108 and 913 mV were visible on the voltammogram. By contrast, iron (II) solution showed only one broad peak at −74 mV of low current.

### 2.3. Deoxyribose Degradation Assay

**Figure 3:**
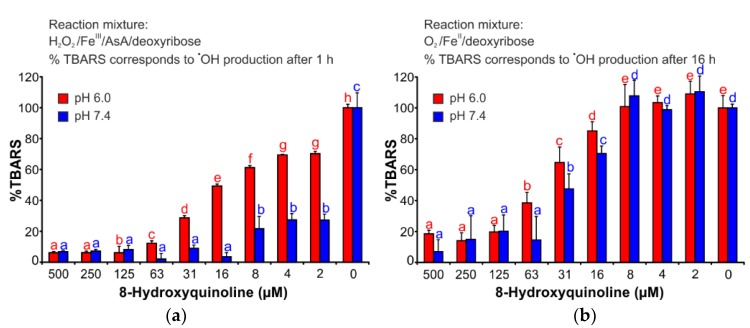
Antioxidant effects of 8HQ in (**a**) deoxyribose degradation assay and (**b**) iron (II) autoxidation assay. The bars are the means of three replications (±S.D.). Letters a–h in subfigure (**a**) and a–e in subfigure (**b**) indicate significance levels (95% Duncan’s post hoc test). AsA: ascorbic acid; TBARS: thiobarbituric acid reactive species; S.D.: standard deviation.

This assay explores the capability of various substances to inhibit the degradation of 2-deoxyribose by hydroxyl radicals, which are generated by the iron-catalyzed Fenton reaction [17]. Figure 3a (Table S1) shows that the antioxidant activity of 8HQ depended strongly on the pH of the reaction solution. 8HQ was a more efficient antioxidant at pH 7.4 than at pH 6.0. Nevertheless, 8HQ demonstrated an apparent ability to inhibit the oxidative degradation of 2-deoxyribose by hydroxyl radical.

### 2.4. Iron (II) Autoxidation Assay

This assay offers the possibility of investigating the effects of various substances on ROS production that is specifically caused by the autoxidation of iron (II) [18]. 8HQ efficiently affected the reactions that decompose 2-deoxyribose (Figure 3b, Table S2). However, in this assay, the effect was less dependent on pH compared to the deoxyribose degradation assay. Nonetheless, the results showed that 8HQ is an efficient antioxidant and it was able to protect 2-deoxyribose against ROS attacks induced by iron (II) autoxidation.

### 2.5. Brine Shrimp Mortality Assay

**Figure 4:**
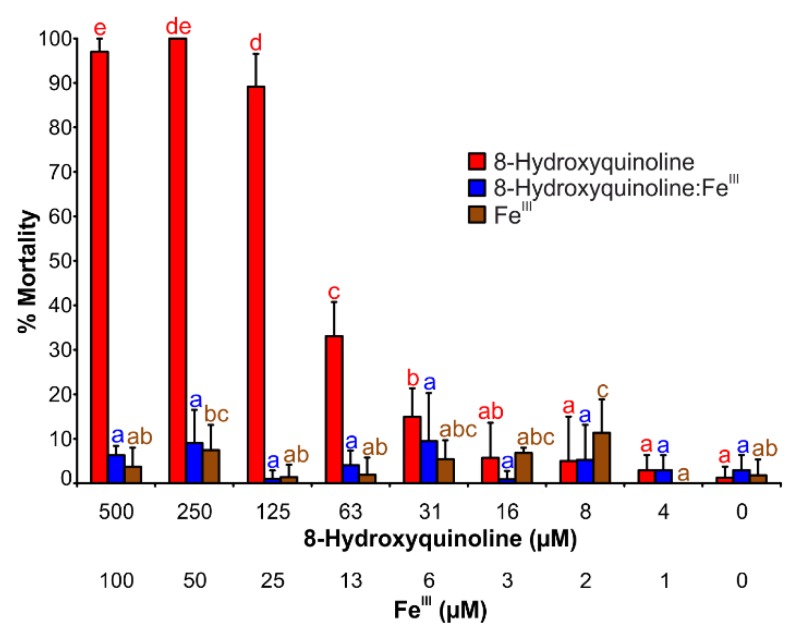
Brine shrimp (*Artemia salina* L.) mortality caused by 8HQ in free form and as Fe^III^−8HQ complexes. The upper scale designates the concentration of 8HQ, the lower one the concentration of iron (III) in the tested solution. The bars represent means of eight replicates (±S.D.). The different significance levels were determined by Duncan’s post hoc test (95%) and are indicated by the letters a–e above the error bars.

The larvae of the invertebrate crustacean *Artemia salina* L. were very sensitive to 8HQ (Figure 4, Table S3). A concentration of 125 µM caused nearly 100% mortality. Contrary, when 8HQ was a ligand in iron coordination complexes, the mortality decreased dramatically. The observed toxicity values matched those of the control assay and of the iron (III) chloride solution.

## 3. Discussion

Mass spectrometry showed that 8HQ can form various coordination complexes in situ with iron (II) and iron (III). In addition to one iron coordination complexes, also two iron coordination complexes were present in the solution. In these binuclear coordination complexes of 8HQ, the iron atoms were present either in the same or in different oxidation states. However, mass spectrometry detected mainly coordination complexes of 8HQ with iron (III) because the corresponding coordination complexes with iron (II) were electro-neutral. Thus, they were not detectable by mass spectrometry. Nevertheless, the ability of 8HQ to easily form coordination complexes with metals is a major reason for the wide use of 8HQ in analytical chemistry for the spectrophotometric determination of various metals [16].

The results of differential pulse voltammetry (DPV) agreed with those obtained by mass spectrometry. Compared to free iron ions, the redox potential of iron in the coordination complexes with 8HQ shifted to more positive values. However, due to the formation of various different complexes in the analyzed solutions, the iron oxidation peak showed a very broad and asymmetric form. Furthermore, the signal of 8-hydroxyl group of Fe–8HQ coordination complexes appeared at more positive potentials in comparison with free 8HQ. Nevertheless, the voltammograms proved that 8HQ antioxidant properties depend not only on quenching iron’s catalysis of the Fenton reaction but also on the redox activity of the 8-hydroxyl group.

The DPV results corresponded well with those obtained in the deoxyribose degradation and iron (II) autoxidation assays. In both, 8HQ was proved as a potent antioxidant. Especially, in the deoxyribose degradation assay, 8HQ showed stronger antioxidant activities than the flavonoids catechin and quercetin [17,22]. Furthermore, 8HQ was more effective in the iron (II) autoxidation assay than a number of well-known phenolic antioxidants such as the flavonoids quercetin and rutin and the phenolic acids chlorogenic, caffeic, and protocatechuic acid, all of which had been assayed in closely similar arrangements [18]. Contrary to quercetin, at pH 7.4, 8HQ showed no pro-oxidant effects in the iron (II) autoxidation assay. However, at pH 6.0 (i.e., the pH of inflamed tissue), antioxidant effects of 8HQ are still possible [18]. The antioxidant ability of 8HQ probably varies due to an extension of the aromatic system that can stabilize semi-quinone radical [23] and protonization of the heterocyclic nitrogen atom and 8-hydroxyl group.

The results of brine shrimp toxicity assay agreed with the results of other published bioassays of 8HQ, especially with those carried out with various microorganisms [10]. 8HQ is well-known as an antimicrobial agent and its derivatives are currently used in medicine, predominately in dermatology [7,8,24]. In the presence of iron ions [10], the antimicrobial activity of 8HQ decreased.

Brine shrimp larvae are often used in ecotoxicology for testing environmental pollutants [20]. However, these larvae are also sensitive to various ROS [25,26] and thus represent good sensors for ROS development. Therefore, we performed brine shrimp mortality assays to explore the possible pro-oxidant effects of the 8HQ complexes with iron as possible ROS producers. Only the free 8HQ caused apparent toxicity. By contrast, the toxicity disappeared in the presence of iron ions in the tested solution. A ratio of 5:1 of 8HQ to iron (III) was chosen to prevent complex formation of iron with phosphate anions of the buffer.

The Fe−8HQ coordination complexes can diffuse easily through cellular membranes due to the increased liposolubility of the coordination complexes. However, 8HQ cannot serve as a good siderophore because of the high stability of its coordination complexes with iron [27]. The long-term iron deficiency caused by 8HQ increased ROS levels and a non-autolytic programmed cell death in plants [27]. In contrast, the brine shrimp mortality assay detected no toxicity of Fe^III^–8HQ coordination complexes. A longer duration of the assay probably would have produced such results.

Our results provided support for the rational background of 8HQ use as a promising agent for neurodegenerative disease treatment, such as Alzheimer’s or Parkinson’s disease. These diseases are well-known to be accelerated by the accumulation of “poorly liganded” iron in the brain tissue that results in increased ROS production [11], especially in the case of damaged blood−brain barrier (BBB) [28]. Our investigation showed that 8HQ is a potent coordination complex-forming agent on the one hand, and an excellent ROS scavenger on the other. Both of these 8HQ properties may contribute to inhibition of the mentioned neurodegenerative processes.

## 4. Materials and Methods

### 4.1. Chemicals

The used chemicals were purchased from Sigma-Aldrich (Schnelldorf, Germany). Water was of Milli-Q quality (Milli-Q Advantage A10 System, Milllipore SAS, Molsheim, France).

### 4.2. Mass Spectrometry

Direct infusion nano-electrospray ionization mass spectrometry was carried out in positive ionization mode on a Thermo Electron LTQ-Orbitrap XL mass spectrometer equipped with a nano electrospray ion source (ThermoFisher Scientific, Bremen, Germany) and operated under Xcalibur software 2.2 (ThermoFisher Scientific, Bremen, Germany) as described by Kubicova et al. [29]. Theoretical masses and characteristic iron isotopic patterns were calculated by Xcalibur software 2.2 (ThermoFisher Scientific, Bremen, Germany).

### 4.3. Differential Pulse Voltammetry

The detailed procedures are described elsewhere [30]. For records of differential pulse voltammograms, a three-electrode system, µAutolab PGSTAT type III (EcoChemie Inc., Utrecht, The Netherlands), was used. A glassy carbon electrode (3 mm of diameter) served as a working electrode, a platinum wire as a counter electrode, and Ag/AgCl (saturated aqueous solution of KCl) as a reference electrode.

The electrochemical experiments were adjusted as follows: The effective scan rate of the voltammetry was 21 mV/s, modulation time was 0.05 s, modulation amplitude was 25 mV, and the scan potential was from −0.250 to +1.200 V. The solution of supporting electrolyte was degassed buffer (0.1 M phosphate buffer pH 7.4; buffer ionic strength 1 M, adjusted by K_2_SO_4_). The voltammograms were recorded for various metal-to-ligand ratios.

### 4.4. Deoxyribose Degradation Assay

The “site-specific” deoxyribose degradation assay (reaction mixture H_2_O_2_/FeCl_3_/ascorbic acid) was performed as described in detail elsewhere [17]. The used buffers were aqueous solutions of KH_2_PO_4_/KOH (30 mM, pH 7.4) and KH_2_PO_4_/H_3_PO_4_ (30 mM, pH 6.0).

### 4.5. Iron (II) Autoxidation Assay

The procedures and reaction mechanisms were published by Chobot et al. [18]. The aqueous solutions of KH_2_PO_4_/KOH (30 mM, pH 7.4) and KH_2_PO_4_/H_3_PO_4_ (30 mM, pH 6.0) were used as buffers.

### 4.6. Brine Shrimp Mortality Assay

For each experiment, 0.5 g of *Artemia salina* cysts were hatched in 25 mL of buffered saline aqueous solution (pH 7.4). Since larvae (nauplii) are extremely sensitive to any quick pH change of the cultivation solution, the experiment was carried out in aqueous solution of buffered saline to stabilize pH (g per 1 L: 8.0 NaCl, 0.2 KH_2_PO_4_, 1.15 Na_2_HPO_4_, 0.2 KCl). For hatching, illumination was performed with a 60 W lamp from a distance of 40 cm for 1 h. The hatched larvae were transferred to an incubator (±25.0 °C).

One hundred μL of the stock solution was serially diluted in 96-well microplates, and 50 µL of suspension of 24-h old larvae (6–30 larvae) were added. The stock solution was prepared by mixing a buffered solution of the tested compound (500 µM) with aqueous solution (100 µM) of iron (III) chloride (1:1 *v*/*v*). The ligand-to-metal ratio was kept at 5:1 to decrease competition of other ligands present in the solution, especially phosphates. The tested iron ions were added in the assay system in their most stable valent state (Fe^III^).

After 24 h, the wells were scored visually for dead animals (larvae without any movement for at least 10 s) by using a stereomicroscope. Fifty µL of 0.1 M HCl were used to kill all animals. In a second scoring survey, all animals were counted for each well to calculate the number of surviving larvae for each concentration tested, as required for a quantal biological assay. The negative control was the buffered saline solution without the test compound. One set of experiments was performed with 8HQ without the addition of iron (III) chloride ions and another experimental set was carried out in solutions of iron (III) chloride without the addition of 8HQ.

## Figures and Tables

**Table 1:** The main signals of ^56^Fe−8HQ (L) coordination complexes in the solutions analyzed by nano-ESI−MS; a positive ionization mode.

			L:Fe^II^ 2:1		L:Fe^III^ 2:1	
Composition	Formula	*m*/*z* Calculated	*m*/*z* Found	Δ (ppm)	*m*/*z* Found	Δ (ppm)
[L + H]^+^	[C_9_H_8_NO]^+^	146.0600	146.0594	−0.59	146.0593	−0.71
[L + Na]^+^	[C_9_H_7_NNaO]^+^	168.0420	168.0413	−0.71	168.0412	−0.82
[L + Fe^II^ + MeOH–H]^+^	[C_10_H_10_ FeNO_2_]^+^	232.0055	232.0044	−4.76	−	−
[3L + Fe^II^ + Fe^III^–3H]^2+^	[C_27_H_18_ Fe_2_N_3_O_3_]^2+^	272.0018	272.0005	−4.86	−	−
[2L + Fe^III^–2H]^+^	[C_18_H_12_ FeN_2_O_2_]^+^	344.0243	344.0223	−5.87	344.0219	−6.98
[3L + Fe^III^–2H]^+^	[C_27_H_19_ FeN_3_O_3_]^+^	489.0770	489.0743	−5.54	489.0740	−6.29
[3L + Fe^III^ + Na–3H]^+^	[C_27_H_18_ FeN_3_NaO_3_]^+^	511.0590	511.0559	−5.93	511.0557	−6.44
[4L + Fe^II^ + Fe^III^–4H]^+^	[C_36_H_24_ Fe_2_N_4_O_4_]^+^	688.0491	688.0454	−5.30	688.0451	−5.84
[5L + 2Fe^III^–5H]^+^	[C_45_H_30_ Fe_2_N_5_O_5_]^+^	832.0940	832.0897	−5.16	832.0893	−5.74
[6L + 2Fe^III^ + Na–6H]^+^	[C_54_H_36_ Fe_2_N_6_NaO_6_]^+^	999.1287	999.1239	−4.86	999.1228	−5.99

## Supplementary Materials

The following are available online at http://www.mdpi.com/1422-0067/19/12/3917/s1.

## Abbreviations

AsA	Ascorbic acid
BBB	Blood−brain barrier
DPV	Differential pulse voltammetry
8HQ	8-Hydroxyquinoline
L	Ligand
Nano-ESI−MS	Nano-electrospray−mass spectrometry
ROS	Reactive oxygen species
S.D.	Standard deviation
TBARS	Thiobarbituric acid reactive species
