# Supplementary material for: Antioxidant Properties and the Formation of Iron Coordination Complexes of 8-Hydroxyquinoline
Source: Int J Mol Sci. 2018 Dec 7;19(12):3917. doi: 10.3390/ijms19123917 (PMC6321042; doi:10.3390/ijms19123917)
Supplement: Supplementary file 1 [file ijms-19-03917-s001.pdf]

International Journal of
Molecular Sciences

MDPI

Supplementary Materials

Table S1. Antioxidant effects of 8HQ in deoxyribose degradation assay (means of 3 replicates, ±S.D., standard deviation).

H_2O_2/Fe^{III}/AsA/deoxyribose Incubation time 1h					
	pH 6.0			pH 7.4	
[μM]	%TBARS	S.D.	[μM]	%TBARS	S.D.
500	6	1	500	7	1
250	6	1	250	7	1
125	6	4	125	8	3
63	12	2	63	2	4
31	29	1	31	9	2
16	49	1	16	4	3
8	61	1	8	22	8
4	70	0	4	27	4
2	70	2	2	27	4
0	100	2	0	100	10

Table S2. Antioxidant effects of 8HQ in iron(II) autoxidation assay, (means of 3 replicates, ±S.D., standard deviation).

O_2/Fe^{II}/deoxyribose Incubation time 16h					
	pH 6.0			pH 7.4	
[μM]	%TBARS	S.D.	[μM]	%TBARS	S.D.
500	19	2	500	7	8
250	14	5	250	15	15
125	20	4	125	20	11
63	39	7	63	15	15
31	65	10	31	48	10
16	85	6	16	71	5
8	100	14	8	108	10
4	103	4	4	99	3
2	109	8	2	111	13
0	100	8	0	100	2

Table S3. Brine shrimp (*Artemia salina* L.) mortality caused by 8HQ in free form and as Fe(III)–8HQ complexes, (mortality means of 8 replicates, ± S.D., standard deviation).

Brine shrimp mortality Incubation time 24h								
	$FeCl_3$			8HQ			$FeCl_3$:8HQ	
**[μM]**	**%Mortality**	**S.D.**	**[μM]**	**%Mortality**	**S.D.**	**[μM]**	**%Mortality**	**S.D.**
100	4	4	500	97	6	100:500	6	2
50	7	6	250	100	0	50:250	9	7
25	1	3	125	89	7	25:125	1	2
13	2	4	63	33	8	13:63	4	3
6	5	4	31	15	6	6:31	10	11
3	7	1	16	6	8	3:16	1	2
2	11	7	8	5	10	2:8	5	8
1	0	0	4	3	3	1:4	3	3
0	2	4	0	1	3	0:0	3	3
